# Identification of pathogenic genes associated with CKD: An integrated bioinformatics approach

**DOI:** 10.3389/fgene.2022.891055

**Published:** 2022-08-11

**Authors:** Mohd Murshad Ahmed, Zoya Shafat, Safia Tazyeen, Rafat Ali, Majed N. Almashjary, Rajaa Al-Raddadi, Steve Harakeh, Aftab Alam, Shafiul Haque, Romana Ishrat

**Affiliations:** ^1^ Centre for Interdisciplinary Research in Basic Sciences, Jamia Millia Islamia, New Delhi, India; ^2^ Department of Biosciences, Faculty of Natural Sciences, Jamia Millia Islamia, New Delhi, India; ^3^ Department of Medical Laboratory Sciences, Faculty of Applied Medical Sciences, King Abdulaziz University, Jeddah, Saudi Arabia; ^4^ Community Medicine Department, Faculty of Medicine, King Abdulaziz University, Jeddah, Saudi Arabia; ^5^ King Fahd Medical Research Center, and Yousef Abdullatif Jameel Chair of Prophetic Medicine Application, Faculty of Medicine, King Abdulaziz University, Jeddah, Saudi Arabia; ^6^ Research and Scientific Studies Unit, College of Nursing and Allied Health Sciences, Jazan University, Jazan, Saudi Arabia

**Keywords:** chronic kidney disease (CKD), DEGs, gene ontology, KEGG, network analysis, molecular docking

## Abstract

Chronic kidney disease (CKD) is defined as a persistent abnormality in the structure and function of kidneys and leads to high morbidity and mortality in individuals across the world. Globally, approximately 8%–16% of the population is affected by CKD. Proper screening, staging, diagnosis, and the appropriate management of CKD by primary care clinicians are essential in preventing the adverse outcomes associated with CKD worldwide. In light of this, the identification of biomarkers for the appropriate management of CKD is urgently required. Growing evidence has suggested the role of mRNAs and microRNAs in CKD, however, the gene expression profile of CKD is presently uncertain. The present study aimed to identify diagnostic biomarkers and therapeutic targets for patients with CKD. The human microarray profile datasets, consisting of normal samples and treated samples were analyzed thoroughly to unveil the differentially expressed genes (DEGs). After selection, the interrelationship among DEGs was carried out to identify the overlapping DEGs, which were visualized using the Cytoscape program. Furthermore, the PPI network was constructed from the String database using the selected DEGs. Then, from the PPI network, significant modules and sub-networks were extracted by applying the different centralities methods (closeness, betweenness, stress, etc.) using MCODE, Cytohubba, and Centiserver. After sub-network analysis we identified six overlapped hub genes (RPS5, RPL37A, RPLP0, CXCL8, HLA-A, and ANXA1). Additionally, the enrichment analysis was undertaken on hub genes to determine their significant functions. Furthermore, these six genes were used to find their associated miRNAs and targeted drugs. Finally, two genes CXCL8 and HLA-A were common for Ribavirin drug (the gene-drug interaction), after docking studies HLA-A was selected for further investigation. To conclude our findings, we can say that the identified hub genes and their related miRNAs can serve as potential diagnostic biomarkers and therapeutic targets for CKD treatment strategies.

## 1 Introduction

Chronic kidney disease (CKD) is considered among the major types of nephrosis across the globe, with a successive increase in its associated patients in recent years (Epstein et al., 1988; Carpenter and McHugh, 2017). CKD is a highly heterogeneous disease, wherein kidney structure and function are damaged (Webster et al., 2017; Tonelli et al., 2006; De Nicola and Zoccali, 2016; Guo et al., 2019; Wilson et al., 2021). Over recent years, although clinical and experimental studies have provided knowledge on the CKD causes (Lovisa et al., 2016; Hewitson et al., 2017; Lajdová et al., 2016; Johnson and Nangaku, 2016), the underlying mechanisms leading to the progression and development of CKD remain poorly understood. After CKD has been diagnosed, the specific CKD stage is determined. CKD is categorized into a five distinct stages on the basis of the glomerular filtration rate (GFR), i.e., G1 (GFR ≥ 90 ml/min/1.73 m^2^), G2 (GFR 60–89 ml/min/1.73 m^2^), G3a (45–59 ml/min/1.73 m^2^), G3b (30–44 ml/min/1.73 m^2^), G4 (15–29 ml/min/1.73 m^2^), and G5 (Kidney International Supplements, 2013). The early CKD stages (Epstein et al., 1988; Carpenter and McHugh, 2017), show few symptoms, and the disease is not detected till it reaches later stages (Rajapurkar et al., 2012). The rate of morbidity and mortality increases with the succession of CKD stages. Particularly adults suffering from CKD have an increased risk of hospitalization due to infections (Dalrymple et al., 2012). The mechanisms linking immune function and kidney function in earlier stages remain poorly understood. CKD entails the slow but regular loss of kidney function and leads to late-stage renal diseases. Transcriptomics is considered a promising strategy for the selection and detection of biomarkers as well as for monitoring the activity of diseases (Szabo and Devlin, 2019). Microarray technologies facilitate the explication of mRNA profiles related to human disease thereby providing a comprehensive and balanced approach for analyzing the processes of disease systematically (Hu et al., 2016). Recent gene expression studies have been successfully carried out on various diseases, such as cancer (Yang et al., 2018; Liu et al., 2015; Zhai et al., 2017), angiocardiopathy (Yan, 2018; Guo et al., 2018), asthma (Modena et al., 2017; Liu et al., 2019), etc. to identify early diagnostic biomarkers. In this context, an in-depth study of the CKD-associated mechanisms is required in order to understand its underlying pathophysiology, which is crucial to identify predictors as well as the therapeutic targets of the disease. Recently, a study has been conducted on CKD gene expression profiles which have identified some of the differentially expressed genes (DEGs) implicated in the development and progression of this disease (Nandakumar et al., 2017). However, we performed an integrated analysis on some of the other unexplored gene expression profiles of CKD. Thus, our identified DEGs show discrepancies with the previous study results due to heterogeneity in CKD cases and control subjects.

The molecular components in human cells are not functionally independent, i.e., interdependent, which means that a particular disease or syndrome is due to the consequence of perturbations of complex intracellular and intercellular interactions. With the enlargement and progression of the network biology field, many potential key genes related to diseases along with their improved drug targets have been identified (Barabási et al., 2011). Thus, this emerging field of network medicine methodically explores drug targets, biomarkers, or key genes of the network through the identification of modules and pathways, therefore, serving as a platform for improved diagnosis, prognosis, and the treatment of complex diseases (Silverman et al., 2020; Conte et al., 2020; Fiscon et al., 2018; Paci et al., 2021).

The present study reports the bioinformatics analysis of the Gene Expression Omnibus (GEO) database available at NCBI (National Center for Biotechnology Information). At first, we applied the meta-approach to CKD patients and healthy controls and retrieved data, to identify the signature DEGs. A total of five gene expression profiling datasets based on CKD (GSE15072, GSE23609, GSE43484, GSE62792, and GSE66494) were selected for the present analysis to screen the DEGs. The overlapped DEGs among all datasets were proceeded to perform the functional enrichment analysis to explore the molecular mechanisms associated with CKD. Then, we carried out the protein-protein interaction (PPI) network analysis to reveal the potential hub genes for CKD. From the PPI network, the modules of interest and hub genes in each module were identified and displayed using Cytoscape. Furthermore, molecular docking of CXCL8 and HLA-A genes was employed with a common drug Ribavirin. Our findings established a reliable biomarker for further research, which may provide a further understanding of the potential molecular mechanisms associated with CKD.

## 2 Materials and methods

### 2.1 The analysis of chronic kidney disease microarray datasets

**FIGURE 1:**
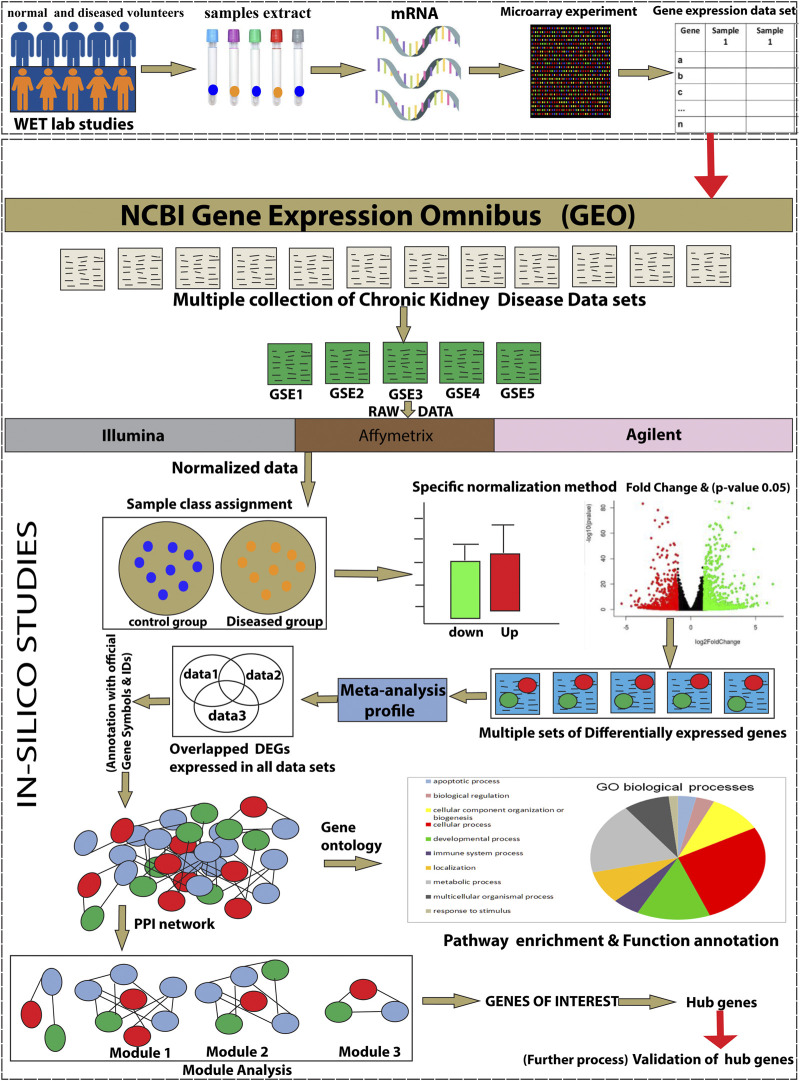
The schematic representation of the present study workflow through an integrative network-based approach.

The workflow of the integrative network-based method used for the present analysis is illustrated in Figure 1. The gene expression datasets (GSE15072, GSE23609, GSE43484, GSE62792, and GSE66494), consisting of normal (healthy control) and treated (diseased patient) samples, were downloaded from the NCBI GEO database (https://www.ncbi.nlm.nih.gov/geo/). The criteria for selection of data in the NCBI website search bar were made using keywords, such as “CKD,” “chronic kidney disease,” and human (Organism). Subsequently, the data obtained from these five datasets were used for the present analysis**.** The details of the retrieved GSE series are mentioned in Table 1.

**TABLE 1:** The details of the present analysis GSE series.

Series	TS	N	D	UR	DR	FC	I	C	Year	Platform	Author
GSE15072	29	8	21	51	38	2	CKD	Italy	2009	GPL-96	Palo Pontrelli
GSE23609	24	7	17	219	189	0.5	CKD	USA	2010	GPL-6454	Persis P. wadia
GSE43484	6	3	3	134	136	0.5	CKD	Sweden	2013	GPL-571	Elham Dadfar
GSE62792	18	6	12	352	262	0.5	CKD	Srilanka	2018	GPL-10558	D. N. Magana
GSE66494	61	8	53	102	325	0.5	CKD	Japan	2015	GPL-6480	Satohiro Masada

a TS, Total samples; N, Normal; D, Disease; UR, Upregulated; DR, Downregulated; FC, Fold change; I, Illness; C, Country.

### 2.2 Data preprocessing, screening, and identification of differentially expressed genes

The retrieved CKD-associated GSE series (the originally obtained data of gene expression datasets) were further processed for normalization through the GEO2R (http://www.ncbi.nlm.nih.gov/geo/geo2r/) tool. It is an interactive web-based tool that allows a user to identify the genes which are differentially expressed across samples. The DEGs were selected by applying default settings, i.e., a *p*‐value < 0.05 after being adjusted by a false discovery rate and |log2FC| > 0.5–2, where FC represents fold change. The linear model analysis of the microarray data package (LIMMA) in R was used to identify DEGs (by defaults in GEO2R) (Ritchie et al., 2015).

The obtained genes (upregulated and downregulated) from the meta-analysis were utilized for the construction of the PPI network. In the process of analysis of DEGs, the Benjamini-Hochberg correction method was used to correct the significant *P*-values obtained by the original hypothesis test.

### 2.3 Protein-protein interaction network construction

For further evaluation of the functional interactions among obtained DEGs, the PPI network was constructed from these DEGs using an online database STRING (Search Tool for the Retrieval of Interacting Genes) Version 11.3, of known and predicted protein-protein interactions. These interactions include physical and functional associations, and the data are mainly derived from computational predictions, high-throughput experiments, automated text mining, and co-expression networks. In order to build a native PPI network of the DEGs, the Gene ID/Probe ID genes were mapped to their respective officially gene symbol/gene name, and their associated *p*-value and FC values were retrieved. Subsequently, Cytoscape 3.8.3 was used to visualize and construct the PPI network. In the PPI network, each node represented the gene and edges represented the connection between nodes.

The following properties in the constructed PPI network were analyzed to find out important behaviors of the network:

#### 2.3.1 Degree distribution

In a network study, the degree (k) of node (n) constitutes the total number of connections or links with other nodes (Gursoy et al., 2008).

#### 2.3.2 Betweenness centrality

In a network, a node’s betweenness centrality reflects the importance of the flow of information from one node to another based on the shortest path.

#### 2.3.3 Closeness centrality

In a network, closeness centrality reflects how quickly the information is passing from one node to another (Przulj et al., 2004).

#### 2.3.4 Stress

In a network, stress is represented as the addition of all nearest paths of all node pairs (Shimbel, 1953).

### 2.4 Hub genes detection based on the centrality approach

To date, various centrality measures have been anticipated in the topology-based network to address the difficulty in locating hub nodes. To discover the most influential nodes or hubs in a complex network constitutes a major problem for researchers. The location of an important node or edge in the network analysis is a major challenge and plays a crucial role. For the identification of significant modules (based on centrality measurement), three different software were employed to identify the significant modules in the PPIN network. CytoHubba (version 0.1) (Cytoscape plugin cytoHubba, a user-friendly interface that ranks nodes in a network based on its features) (Chin et al., 2014), Cytoscape plugin, Molecular Complex Detection (MCODE Version 1.31), and CentiServer (a comprehensive resource, web-based application, and the R Package for the centrality analysis) (Jalili et al., 2015).

### 2.5 The gene term enrichment and pathway analysis

Gene ontology is categorized into three indistinguishable terms, i.e., the molecular function (MF), biological process (BP), and cellular component (CC). These terms collectively give researchers a better idea about the functional annotation of genes. In order to reveal the functional difference among DEGs, the extracted DEGs were submitted to three different enrichment analysis tools, i.e., Enrichr (a comprehensive web server that performs the gene set enrichment analysis) (Kuleshov et al., 2016); g:Profiler, a web server that carries out the functional enrichment analysis of the genes; g:Profiler—a web server for functional enrichment analysis and conversions of gene lists (ut.ee); ToppGene (a web portal which rank or prioritize candidate genes on the basis of the similar function of the training gene list).

### 2.6 Elucidation of hub gene-associated miRNAs

After the identification of hub genes, the miRNet 2.0 platform was used to find out the hub gene-associated miRNAs. MiRNet 2.0 is a web-based platform that helps in elucidating the miRNA using the integrative existing knowledge *via* network-based visual analytics (Fan et al., 2016).

### 2.7 The gene-drug interaction analysis and molecular docking studies

The obtained hub genes were mapped onto the Drug-Gene Interaction database (DGIdb http://www.dgidb.org) (Freshour et al., 2021). The mapping was undertaken to identify better potential therapeutic drugs for CKD-associated genes. The drug-gene interaction complex was visualized using Cytoscape (Version 3.8.2) software. ADGI (Drug-Gene Interaction) is defined as a relationship that exists between a therapeutic and genetic variant and has the possibility to cause an effect on a patient’s treatment in response to a drug. The 3D modeled structures of the obtained hub genes, i.e., CXCL8 (PDB ID: 3IL8) and HLA-A (PDB ID: 1AKJ) were obtained from the RCSB PDB database (https://www.rcsb.org/), and the drug structures were taken from the PubChem database. Using ChemBio3D Ultra software, the 2D structures were transformed into 3D structures. Furthermore, the drug 3D structures were exported as pdbqt files for the docking analysis.

### 2.8 The hub genes analysis in kidney cancer

GEPIA (the Gene Expression Profiling Interactive Analysis) (http://gepia.cancer-pku.cn/detail.php) (Tang et al., 2017), a web server, is used to analyze the expression profiles of tumors (such as KIRC, KIRK, etc.) and normal samples included in TCGA (The Cancer Genome Atlas) (http://portal.gdc.cancer.gov/) and GTEx (the Genotype-Tissue Expression) (http://gtexportal.org/home/) databases. The default parameters were taken and the cutoff value was 50%. The sample was selected as the dataset and the hazard ratio (HR) was calculated based on the Cox proportional-hazards model. The 95% CI was not calculated in the present study. For HR, *p* < 0.05 was considered to indicate a statistically significant difference. Additionally, the validation was carried out for the hub genes using box plot analysis and subsequently their pathological stage was determined.

## 3 Results

### 3.1 Identification of chronic kidney disease-associated differentially expressed genes

The microarray expression datasets GSE15072, GSE23609, GSE43484, GSE62792, and GSE66494 were downloaded from the GEO database. The DEGs between controls and the diseased samples were analyzed using the GEO2R tool. Considering the datasets altogether, our analysis revealed a total of 1793 specific DEGs which included 851 upregulated genes and 942 downregulated genes. For each of the datasets, the total number of upregulated and downregulated genes were also analyzed using the adjusted *p* values (*p* < 0.05) and |log_2_FC| > 0.5–2., and was further compared using an online tool (http://bioinformatics.psb.ugent.be/webtools/Venn/) or Venny 2.0.2. The total number of upregulated and downregulated genes in each dataset is as follows: GSE15072 (50 upregulated and 38 downregulated genes); GSE23609 (219 upregulated and 189 downregulated genes); GSE43484 (133 upregulated and 135 downregulated); GSE62792 (351 upregulated and 262 downregulated); GSE66494 (102 upregulated and 324 downregulated). The overlapped genes that were found to be the most common in at least two GSE series were referred to as final DEGs. The identified overlapped upregulated and downregulated genes in at least two CKD series are mentioned in Table 2.

**TABLE 2:** The identified overlapped DEGs associated with CKD.

GSE series overlapped	Common genes count	Downregulated genes
GSE15072, GSE62792, GSE66494	1	SELENBP1
GSE15072, GSE62792	3	SLC4A1, EPB42, ALAS2
GSE15072, GSE66494	3	PDZK1IP1, HBB, HBD
GSE23609, GSE62792	3	AES, CAPN1, ALDOA
GSE23609, GSE66494	3	AQP3, COX4I1, KLF9
GSE43484, GSE62792	4	TGM2, E2F2, FAXDC2, PKNOX1
GSE62792, GSE66494	10	RPLP0, ITLN1, AMY1C, RPS5, PTGDS, RPL9, RPL13, HAGH, RPL37A, HLA-A
**GSE series**	**Common genes count**	**Upregulated genes**
GSE15072, GSE23609	1	CXCL8
GSE15072, GSE62792	1	ANXA1
GSE23609, GSE43484	3	CD79A, ADAM23, IGHD
GSE23609, GSE62792	2	RBM5, THOC1
GSE23609, GSE66494	2	CRYAB, ZMPSTE24
GSE43484, GSE62792	1	STK38

**FIGURE 2:**
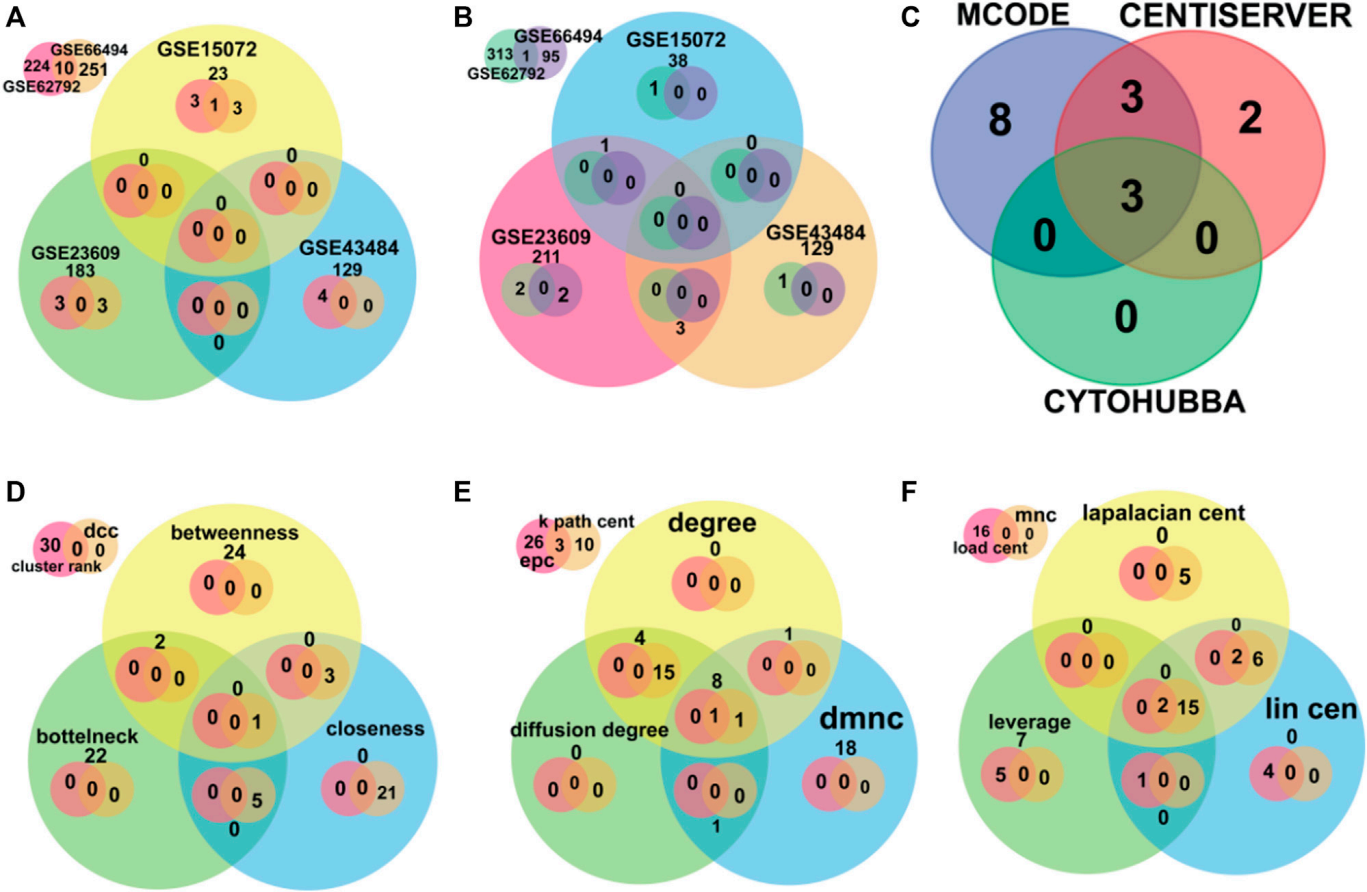
The Venn diagram depicts the overlapped data. **(A,B)** were used for finding overlapped DEGs upregulated and downregulated genes, respectively. **(C)** was used for finding the overlapped hub genes using software MCODE, Centiserver, and Cytohubba. **(D–F)** depict different centralities methods for overlapped genes.

A total of 10 upregulated genes and 27 downregulated genes were proceeded for further investigation as shown in Figure 2.

### 3.2 Construction of the protein-protein interaction network using differentially expressed genes

**FIGURE 3:**
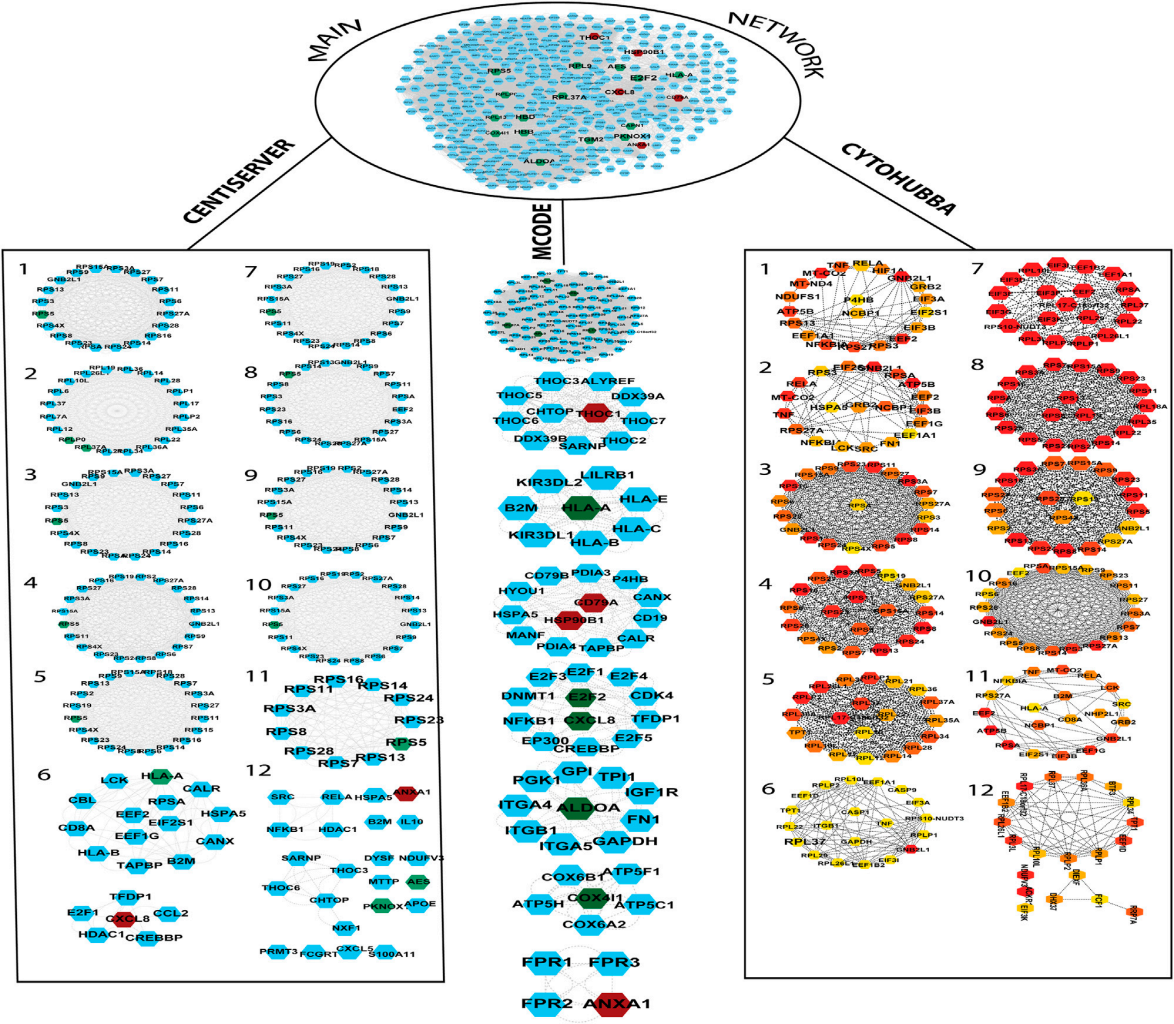
The PPI network was built by the String database. The network contains a total of 322 nodes and 17,239 edges. The PPI network was further divided into subnetworks by MCODE, Centiserver, and Cytohubba. The green color nodes depict downregulated genes, whereas the nodes in red color depict upregulated genes. CytoHubba color coding scheme is based on ranking, wherein the top to bottom gene ranks decline from red to light color.

The gene and its associated products constitute a biological system that forms the basis of a complex network wherein cells interact randomly. During the progression of development of a particular disease, the investigation at the level of gene expression is carried out using the biological information approach. The interrelationship among DEGs was undertaken to identify the overlapping DEGs. A total of 37 genes (including upregulated and downregulated) were submitted to the STRING database, which identified a PPI network that possessed 395 nodes and 17,923 edges. As a variety of pathways and genes are involved in the occurrence and development of a disease, enhanced understanding using network-based approaches is essential for researchers to shed light on disease-related mechanisms. Some crucial genes and signaling pathways perform important biological processes, therefore, they are utilized as therapeutic targets for various diseases. Tracing was applied to the PPI network for the genes of interest finding, which marks that 20 DEGs (out of 37 selected DEGs) represented as each node of the disease-specific gene-interaction network. The interactions of the genes (every single node) formed edges of the network. Then, each significant gene was extracted with its initial neighbors as shown in Figure 3.

### 3.3 Extraction of hub genes based on the centrality approach

It is important to find the most valuable nodes in the complex network. To do this, most researchers find the nodes that are based on centralities methods. In our study, we used 12 different centrality methods in Cytohubba, 12 methods (out of 27) in Centiserver, and one method by MCODE. In our previous research, the most influential nodes in the CVD and CKD network were identified using IVI packages in R. Three genes were traced out 12 modules from the Cytohubba, whereas six genes were traced in MCODE, and nine genes were traced from the Centiserver (Supplementary Table S1). It was observed that three hub genes (RPL37A, HLA-A, and RPS5) were found to be overlapping among three tools/databases (Cytohubba, MCODE, and Centiserver), whereas six genes (RPL37A, HLA-A, RPS5, CXCL8, RPLP0, and ANXA1) were overlapping among two methods/tools/databases (Centiserver and MCODE). The paradigms were exploited for the selection of the top 30 informative genes.

### 3.4 The analysis of gene term enrichment and pathways

It is really important to find, whether a gene is involved in a process and the function of the gene with the location. For these three categories (process, function, and localization), the gene ontology analysis was performed by three different databases (Toppgene, gProfiler, and Enrichr). Various terms were found by these databases, so for the obtained huge data in form of GO terms, we considered only overlapped terms in three categories (MF, BP, and CC). Therefore, out of hundreds of terms, only 12 terms were found to be overlapping, of which two were from the molecular function, six were from the biological process, and four from the cellular process.

The hub genes that showed significant enrichment are listed in Table 3. The pathway analysis of the hub genes was extracted using the Enrichr database as shown in Table 4. Table 3 lists the significantly enriched pathways of hub genes associated with CDK. The significant pathways of hub genes were mainly enriched with Coronavirus disease, cellular senescence, Kaposi sarcoma-associated herpesvirus infection, human cytomegalovirus infection, allograft rejection, cancer, graft-versus-host disease, diabetes mellitus (Type 1), and malaria.

**TABLE 3:** The gene term enrichment analysis of hub genes associated with CKD.

GO term	Category	Function	*p*-value	Count	Gene names
GO:0019843	MF	rRNA binding	9.70E-07	3	RPS5, RPL37A, RPLP0
GO:0003723	MF	RNA binding	6.06E-05	5	RPS5,HLA-A,RPL37A,ANXA1,RPLP0
GO:0006614	BP	SRP-dependent cotranslational protein targeting membrane	2.69E-06	3	RPS5,RPL37A,RPLP0
GO:0006613	BP	Cotranslational protein targeting membrane	3.10E-06	3	RPS5,RPL37A,RPLP0
GO:0045047	BP	protein targeting to ER	4.34E-06	3	RPS5,RPL37A,RPLP0
GO:0000184	BP	Nuclear-transcribed mRNA catabolic process, nonsense-mediated decay	4.03E-06	3	RPS5,RPL37A,RPLP0
GO:0000956	BP	Nuclear-transcribed mRNA catabolic process	2.22E-05	3	RPS5,RPL37A,RPLP0
GO:0010629	BP	Negative regulation of gene expression	3.61E-05	5	RPS5,RPL37A,CXCL8,ANXA1,RPLP0
GO:0005925	CC	Focal adhesion	2.01E-06	4	ANXA1,RPS5,RPL37A,RPLP0
GO:0030055	CC	Cell-substrate junction	2.16E-06		ANXA1,RPS5,RPL37A,RPLP0
GO:0022625	CC	Cytosolic large ribosomal subunit	1.11E-04		RPL37A, RPLP0
GO:0005840	CC	Ribosome	0.018459	1	RPS5

a MF, Molecular function; BP, Biological process; CC, Cellular component. The gene term analysis was performed using different databases Enrichr, gProfiler, and ToppGene.

**TABLE 4:** The pathway analysis of hub genes in CKD.

Term	*p*-value	Count	Genes
Coronavirus disease	2.60E-07	4	CXCL8, RPS5, RPL37A, RPLP0
Ribosome	9.51E-06	3	RPS5, RPL37A, RPLP0
Cellular senescence	8.88E-04	2	CXCL8, HLA-A
Kaposi sarcoma-associated herpesvirus infection	0.001354623	2	CXCL8, HLA-A
Human cytomegalovirus infection	0.001834552	2	CXCL8, HLA-A
Allograft rejection	0.011347288	1	HLA-A
Bladder cancer	0.012238541	1	CXCL8
Graft-versus-host disease	0.012535477	1	HLA-A
Type I diabetes mellitus	0.012832	1	HLA-A
Malaria	0.014908	1	CXCL8

a The pathway analysis was performed using the Enrichr database.

### 3.5 The survival analysis of key genes using Gepia2

**FIGURE 4:**
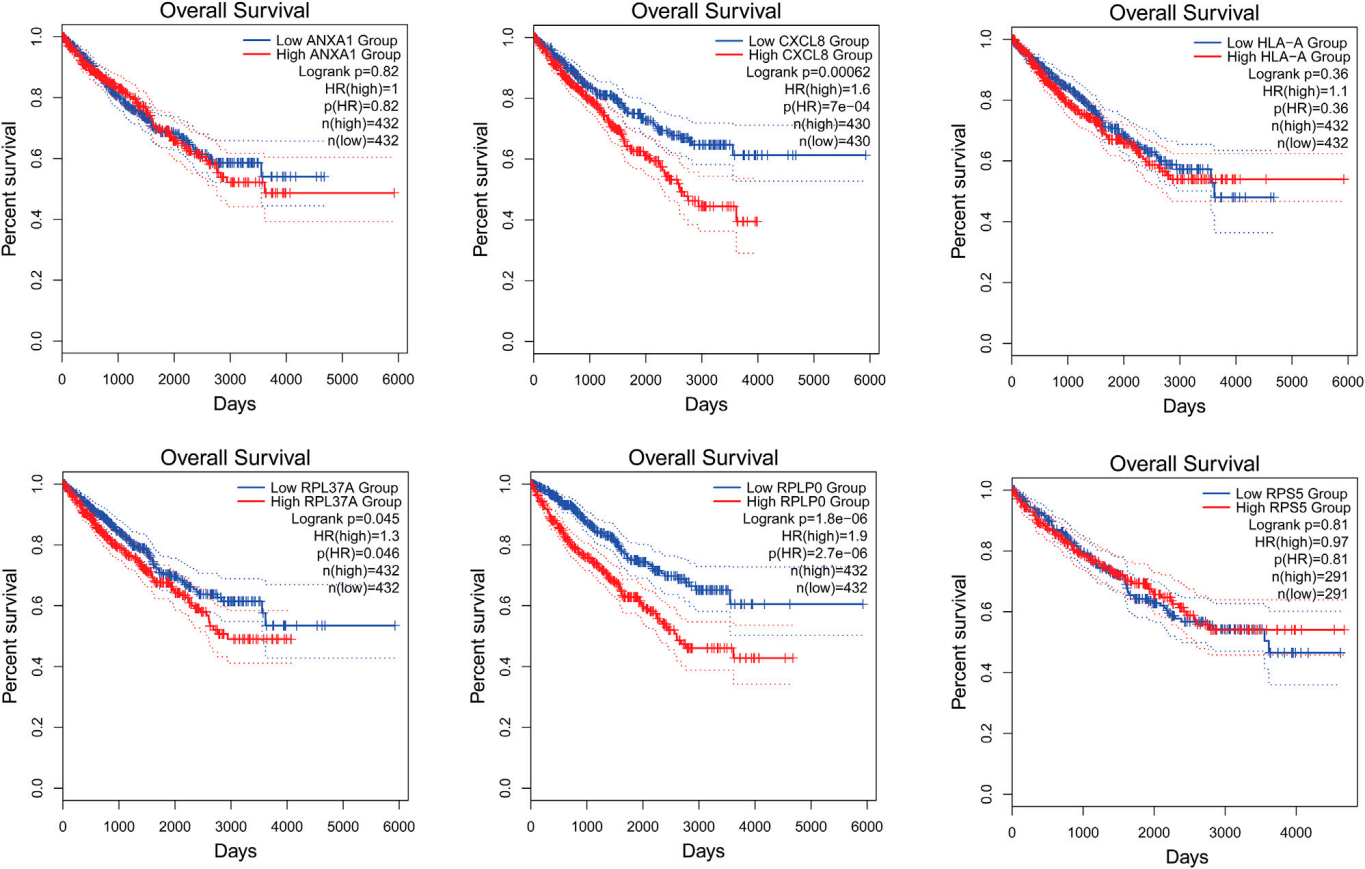
Survival curves of ANXA1, CXCL8, HLA-A, RPL37A, RPLP0, and RPS5 in patients with CKD. Survival curves were used to show the survival ability with time and survival rate.

**FIGURE 5:**
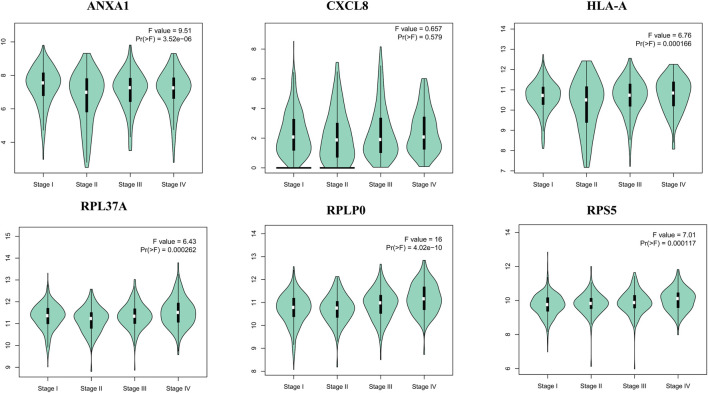
Violin plots of selected hub genes ANXA1, CXCL8, HLA-A, RPL37A, RPLP0, and RPS5 in kidney cancer.

**FIGURE 6:**
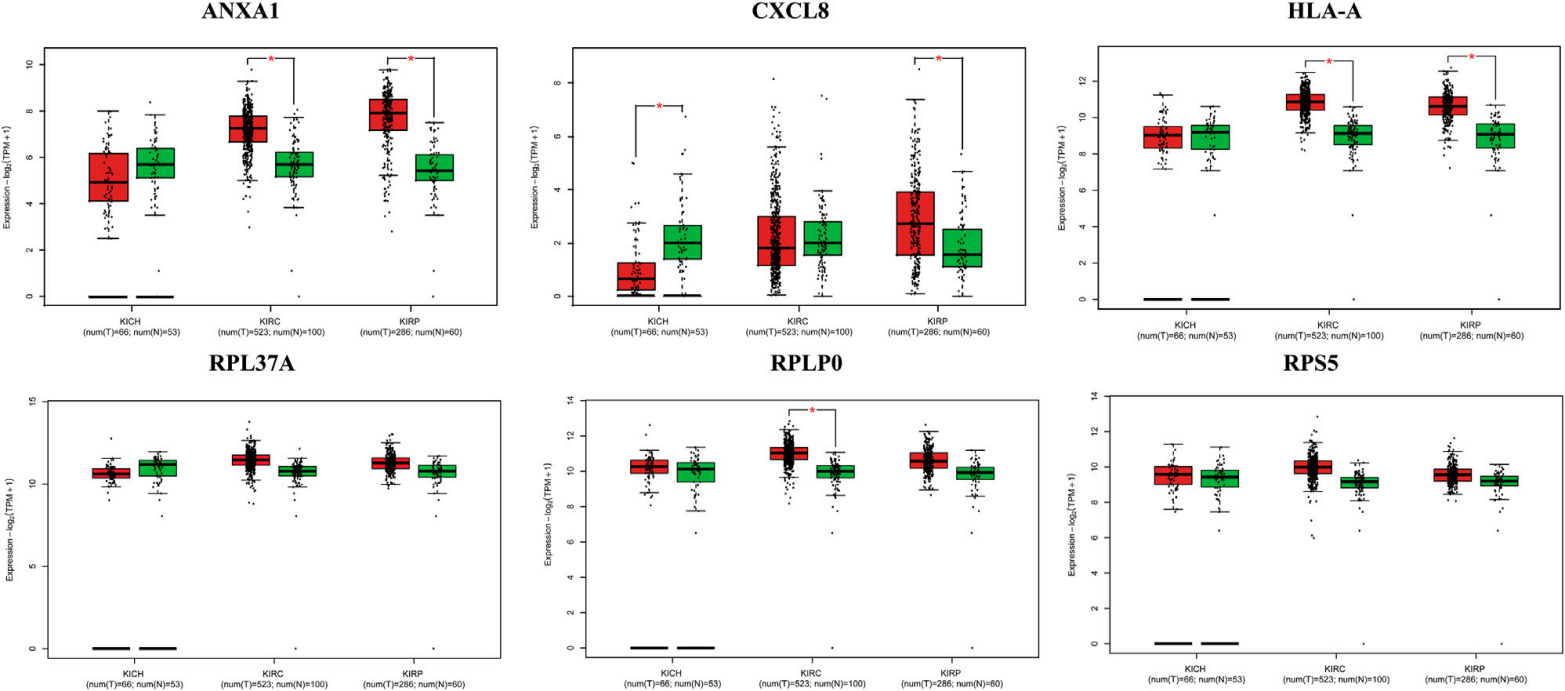
Box plots of selected hub genes ANXA1, CXCL8, HLA-A, RPL37A, RPLP0, and RPS5 in kidney cancer (KIRC, KIRP, and KICH).

The survival analysis of the selected genes (ANXA1, CXCL8, HLA-A, RPL37A, RPLP0, and RPS5) was undertaken. Survival curves are used to show the survival ability with time and survival rate (Figure 4). The relation between hub genes expression and pathological stage in CKD patients was estimated through GEPIA (Figure 5). Further, we utilized GEPIA tool to validate the expression of hub genes using box plot analysis (Figure 6).

### 3.6 Hub genes associated miRNA and gene-drug interaction studies

**FIGURE 7:**
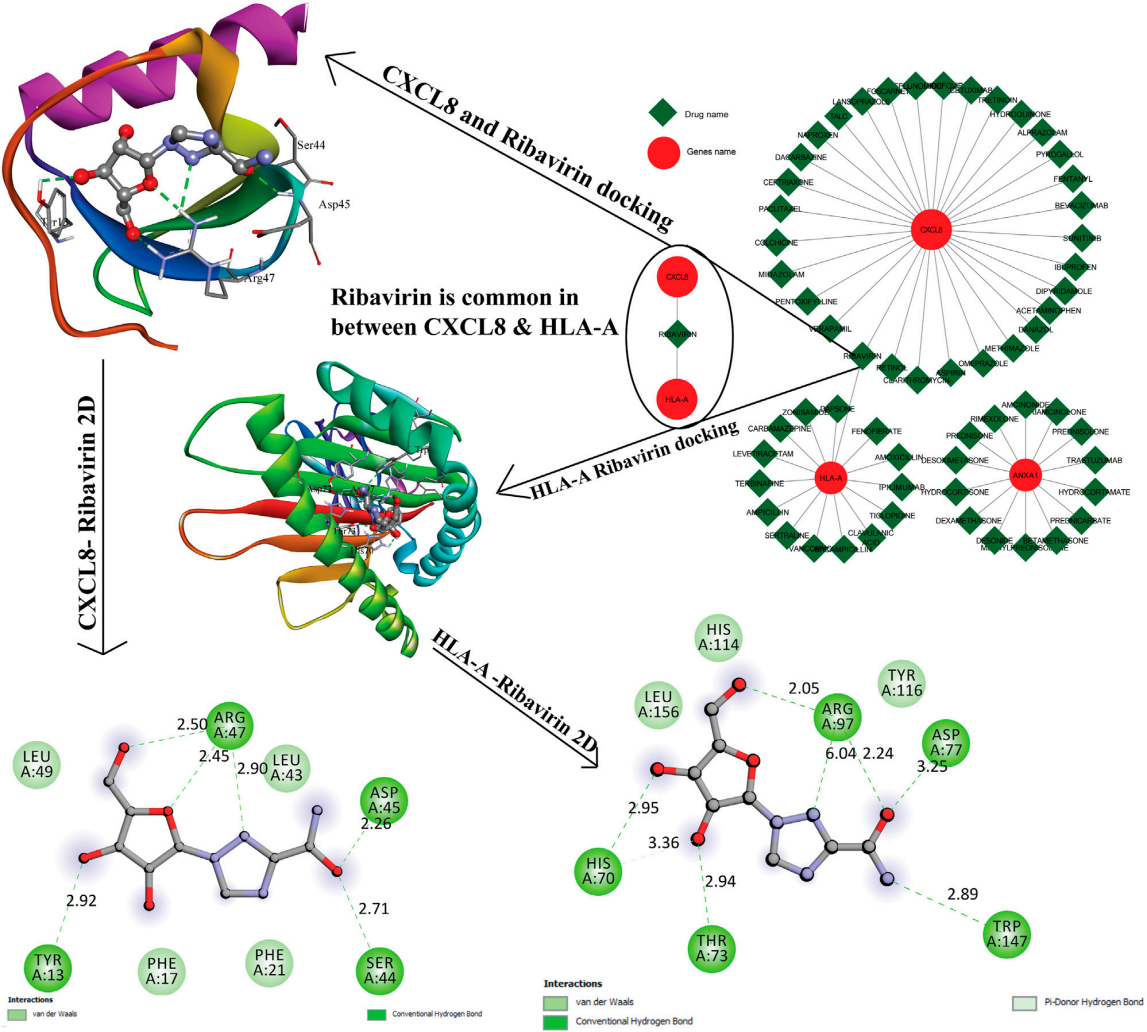
The gene-to-drug interaction network was shown in the figure, the red color nodes are key genes, and the green color nodes their targets drugs. Out of six genes, three genes were making interaction with their target drugs. Ribavirin was a common drug for two genes. This figure shows the 3D interaction and the 2D interaction with ribavirin and CXCL8, HLA-A genes.

The resultant six hub genes (on the basis of centrality approaches) were utilized subsequently to find their target miRNAs see Table 5. For CXCL8 and HLA-A, one miRNA was common hsa-miR-124-3p. They identified six hub genes that were submitted to the DGIdb database and formed a network (the mRNA-Drugs interaction network) with 67 nodes and 61 edges that connected other interacting genes. The network was further analyzed to identify important hub genes with their drug targets. Finally, Ribavirin was identified as the common target for two hub genes (CXCL8 and HLA-A) (Figure 7).

**TABLE 5:** The table shows the list of miRNAs associated with six selected hub genes.

miRNA-target and miRTarbase						
RPLP0	RPS5	CXCL8	HLA-A	ANXA1	RPL37A	
hsa-miR-15b-5p	hsa-miR-23a-3p	hsa-miR-23a-3p	hsa-miR-183-5p	hsa-miR-335-5p	hsa-miR-615-3p	
hsa-miR-221-3p	hsa-miR-15b-5p	hsa-miR-124-3p	hsa-miR-124-3p	hsa-miR-221-3p		
hsa-miR-324-3p	hsa-miR-324-3p	hsa-miR-335-5p	hsa-miR-615-3p			
hsa-miR-16-5p	hsa-miR-16-5p					
hsa-miR-183-5p						
**common**	**RPLP0**	RPS5	**CXCL8**	**HLA-A**	**ANXA1**	**RPL37A**
hsa-miR-15b-5p	✓	✓				
hsa-miR-324b-3p	✓	✓				
hsa-miR-16-5p	✓	✓				
hsa-miR-23a-3p		✓	✓			
hsa-miR-124-3p			✓	✓		
hsa-miR-335-5p			✓		✓	
hsa-miR-221-3p	✓				✓	
hsa-miR-615-3p				✓		✓

Note: The green tick signifies the presence of miRNA, in the respective gene.

## 4 Discussion

CKD affects 8%–16% of the people across the globe. It is one of the major causes leading to death among individuals worldwide. Early diagnosis and staging, with appropriate management by primary care clinicians are important in reducing the burden of CKD worldwide. In past few years, although clinical and experimental studies have provided knowledge on the CKD causes (Lovisa et al., 2016; Hewitson et al., 2017; Johnson and Nangaku, 2016), the underlying molecular mechanisms that lead to the cause and progression of CKD remain to be explored completely. Studies have documented the complex and significant role played by miRNAs in a variety of human diseases including CKD (Liu et al., 2004; Volinia et al., 2006; Hui et al., 2011). MiRNA belongs to a class of small, noncoding, highly stable RNAs that regulate the mRNA and protein expression. Reports have suggested that miRNAs are associated with the regulation of various biological processes, such as proliferation, cellular differentiation, and metabolism (Carrington and Ambros, 2003; Suárez and Sessa, 2009; Hatley et al., 2010). The progression of CKD has been linked to miRNAs (Ahmed et al., 2022). In light of this, the primary objective of our study was to predict candidate miRNAs and hub genes that are associated with the pathogenesis of CKD. We utilized a global approach for the construction of a network based on centrality that predicted clusters of candidate genes involved in CKD.

In this study, five microarray gene expression series (GSE15072, GSE23609, GSE43484, GSE62792, and GSE66494) were retrieved from the Omnibus GEO database to study the relationship between the CKD gene expression and clinical traits. Specifically, from these five gene chips, a total of 1793 DEGs were screened including upregulated and downregulated genes. Our analysis revealed a total of 1,793 specific DEGs which included 851 upregulated and 942 downregulated genes [GSE15072 (50 upregulated and 38 downregulated genes), GSE23609 (219 upregulated and 189 downregulated genes), GSE43484 (133 upregulated and 135 downregulated), GSE62792 (351 upregulated and 262 downregulated), and GSE66494 (102 upregulated and 324 downregulated) in CKD datasets**.** Then next, we determined the overlapping genes among GEO datasets between the upregulated versus upregulated and downregulated versus downregulated genes. The gene term enrichment analysis revealed that the hub genes were mainly involved in RNA binding, co-translational protein targeting, and mRNA catabolic process activities. After putting it all together, a miRNA–mRNA interaction network was constructed. After the construction of the PPI network, we detected the significant modules/hub nodes in the network. The concept of centrality and its associated algorithms have been widely used in the identification of essential nodes in a distinct network. It is considered a promising approach or principal index in biological networks. Using the centrality approach, six hub genes (RPS5, RPL37A, RPLP0, CXCL8, HLA-A, and ANXA1) were selected from the PPI network and were further investigated.

It is interesting to mention that of these identified hub genes, the three hub genes RPS5 (encodes ribosomal protein S5), RPL37A (encodes ribosomal protein 37A), and RPLP0 (encodes ribosomal protein lateral stalk subunit P0), till date, have not been reported to show correlation to CKD. Annexin A1 (ANXA1), is an anti-inflammatory protein that is encoded by the ANXA1 gene. A study has shown the association of ANXA1 with progression and metastasis of cancer, suggesting its role in regulating tumor cell proliferation (Gastardelo et al., 2014). ANXA1 (as an endogenous mediator) has been demonstrated recently to play an important role in alleviating kidney injury in patients with diabetic nephropathy by resolving inflammation (Wu et al., 2021). It has been suggested that intracellular ANXA1 bond inhibits the activation of transcription factor NF-κB p65 by binding to its subunit, thereby, modulating the inflammatory state. Additionally, ANXA1 was found to be abundantly expressed in renal fibrosis (Neymeyer et al., 2015). CXCL8 encodes a protein IL-8 (interleukin-8), which belongs to the CXC chemokine family and is a major mediator of the inflammatory response. IL-8 is also known to promote tumor migration, invasion, angiogenesis, and metastasis (CXCL8 C-X-C motif chemokine ligand 8 [*Homo sapiens* (human)]—Gene—NCBI (nih.gov). The encoded protein is commonly referred to as. Several studies have documented the role of cytokines in chronic kidney disease (CKD) (Vianna et al., 2013). Nagy et al. (2016) also confirmed the expression of CXCL8 in ESRD/ACRD (end-stage renal disease or acquired cystic renal disease kidney) through an immunohistological analysis. Previous investigation has shown the involvement of human leukocyte antigens (HLA) in chronic kidney disease (CKD) patients (Yamakawa et al., 2014). It was revealed that HLAs could act as markers that might be involved in the development of CKD (Yamakawa et al., 2014). Furthermore, a study has demonstrated the genetic association of HLA with a variety of kidney diseases (Robson et al., 2018). Several studies have reported that besides CXCL8 (Noah et al., 2002), other chemokines including CCL2 (Noah and Becker, 2000), CCL3, and CCL5 (Smyth et al., 2002), are found to be upregulated during RSV infection in the nasal fluids. In line with this, Noah and Becker (2000) reported that the level of these chemokines is found to be elevated during the period of illness (viral shedding). A recent investigation has demonstrated that RPL37A and RPLP0 are among the best reference genes (Asiabi et al., 2020). Ovaries from healthy individuals all well as from patients having OEA (ovarian endometrioid adenocarcinoma), OMA (ovarian mucinous adenocarcinoma), OSPC (ovarian serous papillary carcinoma), and PCOS (polycystic ovary syndrome) were identified with some suitable housekeeping genes including RPL37A and RPLP0. Reference genes or housekeeping genes (HKGs) are very important in normalizing mRNA levels between different samples. Since the use of inappropriate reference gene can lead to undependable results, the selection of suitable one is vital for gene expression studies. Therefore, it can be hypothesized that these hub genes may be potential diagnostic biomarkers and therapeutic targets for patients with CKD.

Subsequently, these resultant hub genes were utilized to find their target miRNAs. Moreover, hsa-miR-124-3p was found to be a common miRNA target for both CXCL8 and HLA-A. MicroRNA-124a-3p is related to tumor progression in certain malignant tumors. Esophageal cancer includes a major subtype, i.e., ESCC (esophageal squamous cell carcinoma). The downregulated expression of miR-124-3p has been reported in ESCC tissues, which was found to be correlated with the inhibition of DNA methyltransferase 1 (Zeng et al., 2019). The previous finding has demonstrated hsa-miR-124-3p as a potential target for the diagnosis and prognosis of hepatocellular carcinoma (Long et al., 2018). Thus, the current study suggested that hsa-miR-124-3p plays a crucial role in CKD development by targeting CXCL8 and HLA-A. The investigation of the regulation among hsa-miR-124-3p and CXCL8, HLA-A may shed light on the knowledge of underlying molecular mechanisms of CKD.

Furthermore, the gene-drug interaction was also investigated in order to identify the hub genes and their associated drugs in the CKD network. It was observed that only three (out of the six hub genes) hub genes interacted with drugs and ribavirin was found to be commonly associated with both HLA-A and CXCL8 genes. Unfortunately, ribavirin is not widely used to treat CKD. It has been suggested that treatment with ribavirin prevented RSV-induced CXCL8 production in epithelial cells of humans (Fiedler et al., 1996). It has been revealed in patients (suffering from chronic HCV infection), that the HLA allele is associated with Peg-IFN plus ribavirin therapy (Farag et al., 2013). Altogether, it can be interpreted that our findings from the presented study can contribute to an improved understanding of the mechanisms underlying CKD and the identified hub genes and miRNAs in our study can serve as targets for CKD treatment approaches.

## 5 Conclusion

The present meta-analysis on CKD mRNA expression datasets might provide clues about the potential biomarkers in CKD. The six hub genes ANXA1, CXCL8, HLA-A, RPL37A, RPL3P0, and RPS5 were significantly expressed in these modules. Furthermore studies are underway to address the specific mechanisms of these hub genes in CKD. A detailed understanding of the roles served by these hub genes may provide insights into CKD, and lead to diagnostic and therapeutic opportunities for patients with CKD. Future external validation studies are required to reproduce our findings and determine whether these identified mRNAs or hub genes may influence the CKD progression.

## Data Availability

The original contributions presented in the study are included in the article/Supplementary Material, further inquiries can be directed to the corresponding author.
